# *Piptochaetium fuscum* (Nees ex Steud.) Barkworth, Ciald., & Gandhi, a new combination replacing *Piptochaetium setosum* (Trin.) Arechav.

**DOI:** 10.3897/phytokeys.35.6622

**Published:** 2014-02-26

**Authors:** Mary E. Barkworth, Ana María Cialdella, Kanchi Gandhi

**Affiliations:** 1 Intermountain Herbarium, Utah State University, 5305 Old Main Hill, Logan, Utah 84322-5305 U.S.A.; 2 Museo Botánico, CC 22- B1642HYD San Isidro, Buenos Aires, Buenos Aires, Argentina; 3 Herbaria, Harvard University, 22 Divinity Avenue, Cambridge, Massachusetts 02138-2020, U.S.A.

**Keywords:** *Stipeae*, *Stipa*, *Piptochaetium*, nomenclature

## Abstract

A new name, *Piptochaetium fuscum*, is provided for a taxon hitherto known as *Piptochaetium setosum* (Trin.) Arechav. Morphological, anatomical, and molecular studies that argue against including *Piptochaetium* in *Stipa*, and hence use of *S. purpurata* (Phil.) Columbus & J.P. Sm., are cited.

## Introduction

In February 2010, Gandhi, in responding to an inquiry from Dr. Travis Columbus, noticed that the name *Piptochaetium setosum* (Trin.) Arechav. was superfluous and illegitimate at the time of publication because its basionym, *Urachne setosa* Trin., was itself superfluous and illegitimate at the time of publication, Trinius having included in it two older and validly published names, *Stipa panicoides* Lam. and *Oryzopsis setacea* Rich. *Stipa panicoides* is the basionym of *Piptochaetium panicoides* (Lam.) Desv., a taxon that is now considered distinct from *Piptochaetium setosum* (Parodi 1944; Cialdella and Arriaga 1998; Peña et al. 2008). In addition, Parodi, who examined the types of all the names involved, stated that the type of *Oryzopsis setacea* was evidently based on the same material as that of *Stipa panicoides* (Parodi 1944, p. 299). Thus neither of the names Trinius treated as synonyms of *Urachne setosa* can be used as the basionym for *Piptochaetium setosum* when this taxon is considered to be distinct from *Piptochaetium panicoides*.

Columbus and Smith have published a new name for the taxon, but they placed it in *Stipa* L. as *Stipa purpurata* (Phil.) Columbus & J.P. Sm. (Columbus and Smith 2010). We strongly disagree with their generic interpretation. *Piptochaetium* J. Presl, as interpreted by Parodi (1944; Parodi and Freier 1945; Thomasson 1978, 1979; Cialdella and Arriaga 1998; Cialdella and Guissani 2002; Cialdella et al. 2007; Jacobs et al. 2007; Barber et al. 2009) has been shown to be morphologically, anatomically, and molecularly distinct from other genera of the Stipeae as well as monophyletic. The purpose of this paper is to provide a valid combination in *Piptochaetium* for the taxon hitherto known as *Piptochaetium setosum* in *Piptochaetium*.

Parodi (1944), in his revision of *Piptochaetium*, listed four synonyms for *Piptochaetium setosum*: *Urachne fusca* Steud. (Steudel 1854) (the basionym of *Piptochaetium fuscum*), *Piptochaetium purpuratum* Phil. (Philippi 1857), *Piptochaetium pallidum* Phil. ex Griseb. (Grisebach 1879), and *Piptochaetium macrocarpum* Phil. (Philippi 1896). In describing *Urachne fusca*, Steudel cited a specimen collected by Cuming near Valparaiso, Chile. Parodi stated that he had examined a specimen in B that Nees had annotated as *Piptatherum fuscum*, Valparaiso, Cuming, Herb. Lindley. He also examined two other specimens, one from K and one from CGE, that were labeled Cuming 453. He stated that all three specimens were identical to each other and to the type material of *Urachne setosa* Trin.

## Results and discussion

We have examined images from each of the CGE and K specimens cited above. We agree with Parodi that they belong to *Piptochaetium setosum* as recognized by Arechavaleta (1896) and Parodi (1944) and, on that basis, present the following new combination:

### Piptochaetium fuscum

(Nees ex Steud.) Barkworth, Ciald., & Gandhi comb. nov.

urn:lsid:ipni.org:names:77136235-1

http://species-id.net/wiki/Piptochaetium_fuscum

#### Basionym.

**Figure 1.**
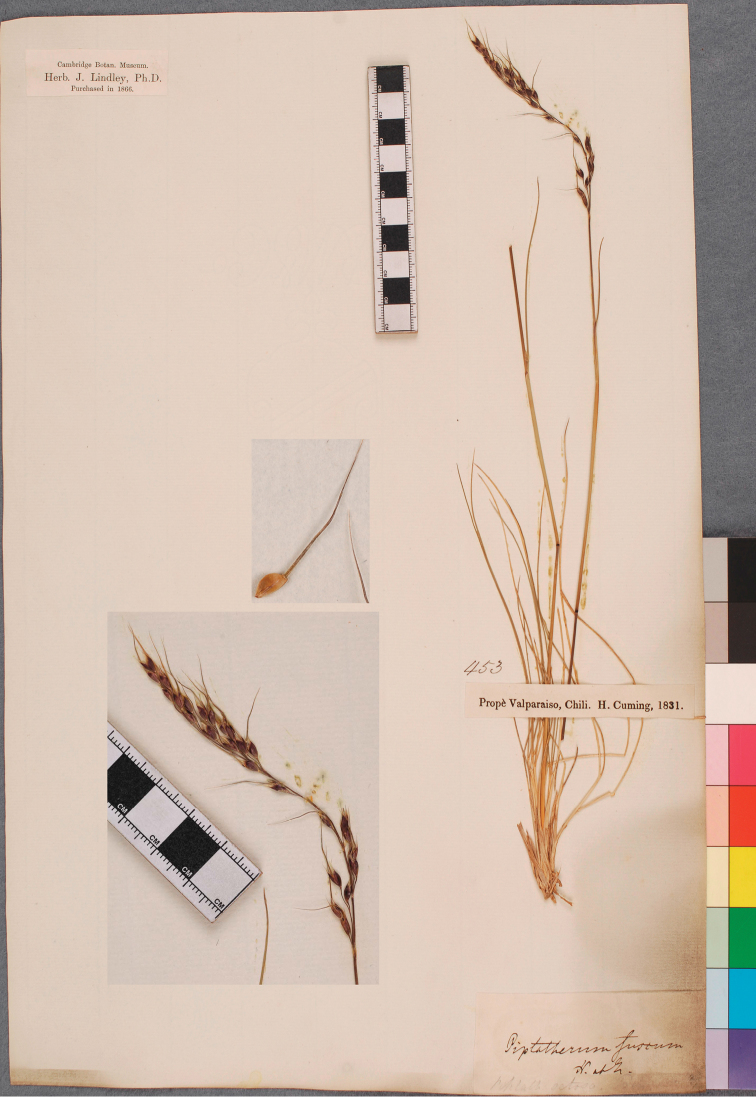
Isotype of *Piptochaetium fuscum* deposited at CGE, the Cambridge University Herbarium, England. Image used with permission.

**Figure 2.**
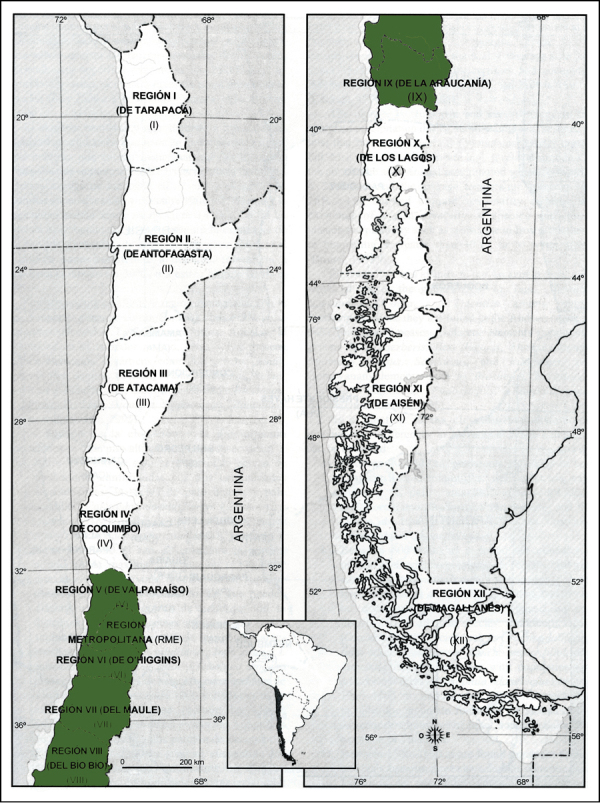
Native distribution of *Piptochaetium fuscum*. Information and base map from Zuloaga et al. (2008), used with permission of Missouri Botanical Garden Press.

*Urachne fusca* Nees ex Steud., Syn. Pl. Glumac. 1(2): 123. 1854 [1855 publ. 2–3 Mar 1854]. Lectotypus: K000433539, Herbarium Hookerianum 1867 (http://specimens.kew.org/herbarium/K000433539), imaginem videmus; Isolectotypi K000433540, Herbarium Benthianum 1854 (http://specimens.kew.org/herbarium/K000433540), imaginem videmus; CGE, Herb. J. Lindley, purchased 1866 (figs 1, 2, 3); “prope Valparaiso, Chili; H. Cuming 453, 1831; Imagines videmus.

The three specimens are from the same gathering and conform to the protologue. We chose K000433539 as the lectotype because it has more material, both reproductive and vegetative, than the other specimens. Columbus and Smith (2010) were forced to base their name on *Piptochaetium purpuratum* Phil. because the name *Stipa fusca* had already been used for an Australian taxon by Hubbard (1925).

Parodi (1944) stated that *Piptochaetium fuscum* grew in central Chile, extending from Valparaiso and Santiago to Valdivia. This statement was confirmed by Zuloaga et al. (2008) who added that it grew at 0-800 m. There are only three South American records with latitude and longitude in the Global Biodiversity Information Facility. They were collected at 37.41S, 72.01W [SI 268952]; 36.48S, 72.71W [BAA 416344], and 36.56S, 72.49W [BAA 416345]. Zuloaga et al. (2008) provide information in terms of Chile’s regions (Fig. 2). The species is also known from one locality in Marin County, California, where it was first collected in 1978 (Consortium of California Herbaria 2014). The origin of the population is unknown. It does not appear to have spread since its introduction.

To determine the conservation status of *Piptochaetium fuscum*, a search should be made for specimens in Chilean herbaria and field work conducted to locate natural populations. Such activities were beyond the scope of our study.

## Supplementary Material

XML Treatment for Piptochaetium fuscum
